# Reply to Yigit, S.; Akinci, B. Comment on “Vázquez-Gandullo et al. Inspiratory Muscle Training in Patients with Chronic Obstructive Pulmonary Disease (COPD) as Part of a Respiratory Rehabilitation Program Implementation of Mechanical Devices: A Systematic Review. *Int. J. Environ. Res. Public Health* 2022, *19*, 5564”

**DOI:** 10.3390/ijerph20064801

**Published:** 2023-03-09

**Authors:** Eva Vázquez-Gandullo, Antonio Hidalgo-Molina, Francisca Montoro-Ballesteros, María Morales-González, Isabel Muñoz-Ramírez, Aurelio Arnedillo-Muñoz

**Affiliations:** 1 Pneumology, Allergology and Thoracic Surgery Department, University Hospital Puerta del Mar, 11009 Cádiz, Spain; 2 Pneumology Department, Hospital Punta Europa, 11207 Cádiz, Spain

We would like to thank you for your interest [1] in our systematic review article about inspiratory muscle training (IMT) in COPD patients [2] and for sharing your point of view on it.

We have been carefully evaluating all the considerations that you have presented to us, and we intend to answer them below in order to resolve any doubts that may have arisen in our article.

First of all, we have been assessing both the quality of the studies and the way of evaluating the quality in our systematic review.

In our review article, we carried out a systematic review of articles following the PRISMA recommendations [3], in which we used different databases independently. To determine the suitability of the studies, we established PICO questions that included: (a) participation of COPD patients, (b) avoidance of duplicity of studies, and (c) implementation of an inspiratory muscle training program, as reflected in the figure number 1 included in the article [2].

The assessment of the quality of the studies was defined according to the following criteria set out in our article [2], mainly the inclusion of original articles, systematic reviews, and meta-analyses, which were less than 10 years old.

Secondly, you emphasized the need to establish an IMT protocol in respiratory rehabilitation programs for COPD patients.

At present, updates to the guidelines for the management of COPD show the growing need for these patients to participate in a respiratory rehabilitation program, which is why we believe it would be ideal to establish a personalized protocol for each COPD patient based on their needs.

However, the implementation of respiratory rehabilitation programs in COPD patients is not recommended by scientific societies such as the British Thoracic Society (BTS) or the Spanish Society of Pneumology and Thoracic Surgery (SEPAR), although the latter recommends the addition of inspiratory muscle training in patients with inspiratory muscle weakness, defined as a maximum inspiratory pressure of less than 60 cm H2O, as reflected in our article [2].

There is also no standardized IMT protocol due to the great heterogeneity in the published studies, with differences in program duration, exercise intensity, number and type of patients included, etc., as shown in Table 1 of our article [2].

Therefore, establishing an IMT protocol would facilitate its application in real life and its incorporation into clinical practice guidelines. 

As for the ideal IMT protocol, we consider that it should be of short duration (8–12 weeks) to avoid losing patients and with high intensity (70–80% of maximum workload) exercises so that patients notice the beneficial effect of this training, which would reinforce compliance on their part [4,5,6].

Finally, you comment on the importance of evaluating the effect on the quality of life of COPD patients studied in the articles presented in our systematic review. 

First, we must mention that the aim of the review was focused on explaining IMT, which devices can be used for its implementation and which of them have shown benefit in patients with COPD.

Considering this and with the limitations mentioned in the article, we have been able to gather the devices, what role they played, and the objectives obtained with each of them. 

Taking the above into account, devices such as Respifit STM [7], SpiroTigerR [8], or FeelBreatheR [9] presented positive results in terms of increased quality of life. 

Systematic reviews such as the one by Beaumont et al. [10], whose objective is focused on verifying the effect of IMT in COPD patients in terms of quality of life, dyspnea, or exercise capacity, confirm the usefulness of the implementation of IMT. The interesting review by Ammous et al. [11] deserves a special mention, which addresses an essential aspect of this topic: Whether the benefit is greater when IMT is associated with a Respiratory Rehabilitation (RR) program or if it is greater when is implemented in isolation. To answer this, new studies will be necessary.

We appreciate all the considerations indicated, and we hope to have resolved all the doubts raised, since we agree on the importance of RR and the benefits that IMT can bring to a selected profile of patients.

## Data Availability

All data are available from the corresponding author upon reasonable request.
